# Rapid Detection of Necrosis in Breast Cancer with Desorption Electrospray Ionization Mass Spectrometry

**DOI:** 10.1038/srep35374

**Published:** 2016-10-13

**Authors:** Alessandra Tata, Michael Woolman, Manuela Ventura, Nicholas Bernards, Milan Ganguly, Adam Gribble, Bindesh Shrestha, Emma Bluemke, Howard J. Ginsberg, Alex Vitkin, Jinzi Zheng, Arash Zarrine-Afsar

**Affiliations:** 1 Techna Institute for the Advancement of Technology for Health, University Health Network, Toronto, ON, M5G-1P5, Canada; 2 STTARR Innovation Center, Princess Margaret Cancer Center, 101 College Street, Toronto, ON M5G 1L7; 3 Department of Medical Biophysics, University of Toronto,101 College Street Suite 15-701, Toronto, ON, M5G 1L7, Canada; 4 Waters Corporation, 34 Maple Street, Milford, MA 01757, USA; 5 Department of Surgery, University of Toronto, 149 College Street, Toronto, ON, M5T-1P5, Canada; 6 Keenan Research Center for Biomedical Science, Li Ka Shing Knowledge Institute, St. Michael’s Hospital, 30 Bond Street, Toronto, ON, M5B-1W8, Canada; 7 Department of Radiation Oncology, University of Toronto, 610 University Avenue, Toronto, Ontario M5G 2M9, Canada; 8 Division of Biophysics and Bioimaging, Ontario Cancer Institute, University Health Network, 610 University Ave, Toronto, ON M5G 2M9, Canada; 9 Institute of Biomaterials and Biomedical Engineering, University of Toronto, 164 College Street, Toronto, ON M5S 3G9, Canada.

## Abstract

Identification of necrosis in tumors is of prognostic value in treatment planning, as necrosis is associated with aggressive forms of cancer and unfavourable outcomes. To facilitate rapid detection of necrosis with Mass Spectrometry (MS), we report the lipid MS profile of necrotic breast cancer with Desorption Electrospray Ionization Mass Spectrometry (DESI-MS) imaging validated with statistical analysis and correlating pathology. This MS profile is characterized by (1) the presence of the ion of *m/z* 572.48 [Cer(d34:1) + Cl]^−^ which is a ceramide absent from the viable cancer subregions; (2) the absence of the ion of *m/z* 391.25 which is present in small abundance only in viable cancer subregions; and (3) a slight increase in the relative intensity of known breast cancer biomarker ions of *m/z* 281.25 [FA(18:1)-H]^−^ and 303.23 [FA(20:4)-H]^−^. Necrosis is accompanied by alterations in the tissue optical depolarization rate, allowing tissue polarimetry to guide DESI-MS analysis for rapid MS profiling or targeted MS imaging. This workflow, in combination with the MS profile of necrosis, may permit rapid characterization of necrotic tumors from tissue slices. Further, necrosis-specific biomarker ions are detected in seconds with single MS scans of necrotic tumor tissue smears, which further accelerates the identification workflow by avoiding tissue sectioning and slide preparation.

Necrosis is a form of cell death caused by factors external to the cell, such as hypoxia, and is often associated with rapidly growing, aggressive forms of cancer in the breast, colon, brain, lung, kidney, and pancreas. Identification of tumor necrosis in breast cancer can provide prognostic information indicating early recurrence or death, and is used in treatment planning^1^. Therefore, necrosis in tumors must be detected during surgery to allow immediate treatment planning once the tumor is encountered. Currently, intraoperative histology can be used to identify necrosis in resected lumpectomy samples. The method uses either a snap frozen slice of the biopsied tissue or a tissue smear prepared on a microscope slide. Morphological signatures of necrosis, such as alterations in the cellular and nuclear membranes, are then used to identify necrotic centers through microscopy. The process can take anywhere between 10–30 min, and requires feedback from a trained pathologist. Due to lengthy processing times, only small samples of tissue are examined during surgery and regional sampling error can lead to necrotic areas being overlooked. Intraoperative assessment of necrosis is often completed while the patient is under general anaesthesia. Therefore, there is a need for a much more rapid analysis platform to allow a larger number of samples to be examined intraoperatively for necrosis compared to what is currently possible.

Mass Spectrometry (MS) is a highly sensitive analytic technique capable of characterizing the chemical content of a sample in milliseconds. MS provides the chemical fingerprint of biological tissue on the basis of the mass-to-charge (*m/z*) ratio of its constituent molecules (MS profiling). Furthermore, MS measurements can be performed in the imaging mode to provide a spatially resolved molecular image of biomarker ions characteristic of the tissue under study (MS imaging). The development of ambient MS techniques such as Desorption Electrospray Ionization (DESI)^2^, where a spray of charged microdroplets of solvent desorbs and ionizes tissue molecules, has allowed identification of cancer on the basis of characteristic lipid MS profiles obtained from the tumor^3^,^4^,^5^,^6^,^7^,^8^,^9^. DESI-MS analysis also allows tumor type identification and grading on the basis of MS lipid profiles unique to each tumor subclass^3^,^5^,^7^,^8^,^10^,^11^,^12^,^13^. Currently, cross-validation with gold standard pathology is needed to interpret DESI-MS images of tumor slices by translating the chemical heterogeneities of the tissue slice, discovered through DESI-MS, to meaningful changes in the tumor structure in that slice. The predictive power of MS profiling demonstrated to date, however, suggests that in combination with robust cancer MS profile libraries, ambient MS with DESI will become a new tool at the disposal of pathologists to aid in rapid intraoperative characterization of cancer. For this to become a reality two requirements must be met. First, the workflow for MS analysis must be optimized to deliver pathology information on timescales that are clinically suitable (less than ten minutes is ideal). Second, a library of cancer DESI-MS spectra needs to be available in order to perform predictions of the tissue state when examining real clinical samples. These constitute two major obstacles that must be overcome to enable the future adoption of MS for clinical pathology^14^.

The spatially non-targeted nature of current MS imaging approaches requires interrogation of the entire surface of the excised tissue even if the information is sought from a sample subsection. Note that a visual inspection of the tissue slice often does not provide sufficient information/contrast to guide the MS operator to the areas suspected of necrosis. However, the structural alterations in the tissue that accompany necrosis alter the optical depolarization rate, allowing tissue heterogeneities to be detected with polarimetric imaging and thus targeted with MS analysis, reducing overall analysis time. We have thus recently reported the process of using polarized light imaging (Mueller Matrix Polarimetry) to spatially target MS analysis^15^. The work described in this manuscript elaborates on this recent work^15^ by further addressing the limitations above as they relate to rapid detection of necrosis in breast cancer with DESI-MS.

We hereby describe a workflow for rapid, DESI-MS analysis of necrotic breast cancer using wide-field polarimetry guidance, a concept previously reported by our group to have utility in rapid MS imaging of margins for infiltrating breast cancer^15^. Murine xenograft tumors of human breast cancer were studied with DESI-MS, statistical analysis and pathology to determine the lipid MS profile of necrotic breast cancer. We then validated the profile by verifying the concordance between assignments of necrosis with DESI-MS and with pathology in a blind test using three independent tumors. We further showed that wide-field tissue polarimetry can indicate necrotic areas for the purpose of guiding DESI-MS profiling, allowing detection of necrosis in a total measurement time of ~2 min, which is faster than current histopathology methods. Further reduction in the analysis time could be achieved through use of tissue smears, which eliminates the processing time required for freezing and slicing. The MS profile of necrosis was detectable from smears with only a single MS scan in ~1 s of collection time. With the use of image guidance or tissue smears, DESI-MS shows promise in delivering a rapid, accurate and objective measure of the presence of necrosis within tumors.

## Materials and Methods

### Animal studies

All animal studies were conducted in accordance with institutional guidelines and approved by the animal ethics and use committee (Animal Use Protocol at the University Health Network, Toronto, Canada). 4 × 10^6^ LM2-4-luc+ triple negative breast cancer cells were inoculated into the right inguinal mammary fat pad of four female Severe Combined Immunodeficiency (SCID) mice. Primary tumors were surgically removed three weeks post-implantation, and the metastatic progression was further monitored for five weeks. At the study end point, Bioluminescence Imaging (BLI) and Magnetic Resonance Imaging (MRI) were performed (Supplementary Figure S1), and tumor nodules were collected for histological examination. Primary tumor (tumor 1), the regrown tumor at the site of inoculation (tumor 2) and metastases in the axillary lymph nodes in the upper limb (“training” tumor and tumor 3) were subsequently frozen on liquid N_2_ vapour and stored at −80 °C.

### *In Vivo* imaging

MRI was performed on a 1 Tesla preclinical MRI scanner (M3, Aspect Imaging) with a 50 × 30 mm mouse body coil and the following parameters: Fast Spin Echo, TE/TR = 55.6 ms/6833 ms, ETL = 16, flip angle = 90°, FOV = 40 × 90 mm, matrix size = 96 × 256, 8 averages, final voxel size 0.35 × 0.35 × 0.42 mm.

BLI was performed on a Xenogen IVIS - 100 Imaging System (Perkin Elmer) as described previously^15^.

### Tissue Sample preparation

Flash frozen tumors were mounted onto a metal specimen holder with a small amount of Tissue-Tek Optimal Cutting Temperature (OCT) compound from Sakura Finetek USA Inc. Serial slices with thicknesses of 10 μm were prepared and stored as described^15^. DESI-MS imaging and histological analysis were performed on identical slices, whereas polarimetry was performed on consecutive tissue sections of the same thickness.

### Polarimetry

10 μm slices of breast cancer tumors mounted on glass slides were subjected to polarimetry measurements using a home-built wide field polarized light imaging system operating in transmission geometry described previously^15^. Mueller matrix at each imaged pixel for the sample was calculated as reported^16^. The polarization parameter of depolarization was extracted using Lu-Chipman Mueller matrix decomposition^17^.

### Laboratory Histology Analysis

Hematoxylin & Eosin (H&E) staining was performed as reported previously^15^. A sequential section was stained for Pan-Cytokeratin (Clone AE1/AE3, Dako) using the Vector ImmPRESS HRP Anti-Mouse Ig (Peroxidase) Polymer Detection Kit (Vector Laboratories) and visualized with DAB (Vector Laboratories). All digital images were captured using a TissueScope 4000 slide scanner (Huron Technologies).

### DESI-MS and DESI-MS imaging experiments

All MS experiments were performed using a Xevo G2XS Quadrupole-Time-Of-Flight Mass Spectrometer (Q-TOF-MS, Waters). The glass slides containing 10 μm slices were mounted on a 2D moving stage, and subjected to DESI-MS imaging in the negative ion mode over the mass range *m/z* 200 to 1000. A 1:1 mixture of acetonitrile and dimethylformamide (both HPLC-MS grade, Sigma Aldrich, Oakville, ON, Canada), containing 150 pg/μL Leucine Enkephalin (*m/z* 554.26) for post-acquisition correction of *m/z* values, was used as the charged spray solvent, and delivered at a flow rate of 1 μL/min. The sprayer-to-surface distance was 1.0 mm, the sprayer to MS inlet tube distance was 5 mm, and incident spray angle was set to 68°. The source parameters were 3.6 kV capillary voltage, 150 °C capillary temperature, and nitrogen spray of 100 psi. In order to acquire DESI-MS images, tissues were raster-scanned at a velocity of 100 μm/s, with a scan time of 1s, and a spatial resolution of 100 μm. High Definition Imaging (HDI) platform version 1.35 (Waters) was used to process the mass spectral data and to generate 2D spatially resolved ion images. Spectra were recalibrated for high mass accuracy using the accurate mass of Leucine Enkephalin present in the solvent spray. Assignments of the lipid biomarker ions in the negative ion mode were made through DESI-MS/MS using LIPID MAPS database or isotopic pattern match, further verified with LC-MS using lipid extract as detailed in Supporting Information.

### Principal Component Analysis

Average spectra from two distinct Regions of Interest (ROI) from viable/necrotic tissue (guided by pathology) from each tumor were defined through the HDI software, from which a list of ions was extracted (i.e. data point). We used *m/z* values to two decimal points. Using the MetaboAnalyst platform (http://www.metaboanalyst.ca), with a mass tolerance of 20 ppm, we corrected for the missing values using Probabilistic PCA. The rationale here was to use an interpolation of data to avoid deletion of columns with missing values. Other means of achieving interpolation available through MetaboAnalyst such as nearest neighbor estimation were also applied, and found not to significantly alter the PCA results. We thus, for consistency’s sake, applied the probabilistic PCA method to all spectra obtained. This resulted in no need to delete columns containing *m/z* mismatch, or ‘missing values’. A typical peak list contained 500 *m/z* values. Normalization by sum (Total Ion Current) and scaling by the Pareto method were performed to address the dependence of the rank of biomarker ions on the intensity as recommended for PCA^18^. However, subjecting our data to PCA using mean-centering method available now through MetaboAnalyst platform did not change the outcome. Scores plots and box plots (indicating ion intensity normalized to TIC) were extracted from the PCA runs, and presented without further modifications^19^,^20^. From the loading plots, *m/z* values that contributed most significantly to the separation were chosen and further studied in terms of changes in intensity. PCA is an unsupervised statistical analysis method widely used in analyzing mass spectrometry images to determine latent features that create heterogeneity within a sample.

### Non-Negative Matrix Factorization (NMF) Analysis

NMF is an unsupervised statistical analysis method that explores the entire MS imaging dataset for similarities in spatial distribution of ions to reveal distinct MS profiles that populate tissue subsections, each possessing different but correlated spatial distributions^21^. The mass spectral matrices can be considered as non-negative quantities exhibiting intensity-dependent noise^21^. Image files generated by the HDI software (waters) from a heterogeneous tumor containing necrotic and viable centers were subjected to 4 component NMF analysis using omniSpect^21^ (http://cs.appstate.edu/omnispect/). Images of spectrally correlated areas along with characteristic mass spectra thereof were generated using this platform and incorporated into the manuscript. NMF analysis provides a map of heterogeneity within a tissue image solely on the basis of the mass spectral information. As such, NMF does not use feedback from pathology.

## Results and Discussion

**Figure 1:**
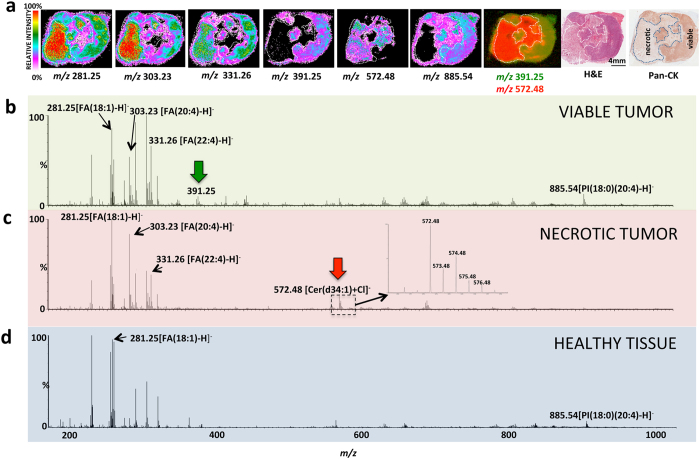
DESI-MS imaging of necrotic breast cancer tumor. (**a**) DESI-MS ion images of the markers detailed in Supplementary Table S1 alongside H&E and Pan-CK immunostained images of the tumor. Here, we also show the merged image of the biomarker ions of *m/z* 391.25 and *m/z* 572.48 that characterize the viable and necrotic tissue, respectively. This merged image delineates the entire shape of the tumor tissue. (**b**) The average DESI-MS spectrum of the viable cancer tissue. (**c**) The average DESI-MS spectrum of the necrotic region with an inset of the enlarged spectrum at *m/z* 572.48 to show its isotopic pattern is characteristic of a chlorinated ion. (**d**) DESI-MS spectrum of healthy mammary fat pad breast tissue, indicating little overlap with the DESI-MS profile of cancerous tissue. The average spectra were obtained from the entire tumor subregions selected as ROI in the HDI software package. The ROI used for averaging are marked with the white dashed lines over the images in (**a**). The known breast cancer biomarker ions of *m/z* 281.25 and 303.23 are more abundant in necrotic tissue. The biomarker ion of *m/z* 331.26 is more abundant in the viable cancer region. The ion of *m/z* 572.48, absent in the viable cancer tissue, was prominent in the necrotic region, and the ion of *m/z* 391.25 present in the viable cancer tissue was absent from necrotic regions.

**Figure 2:**
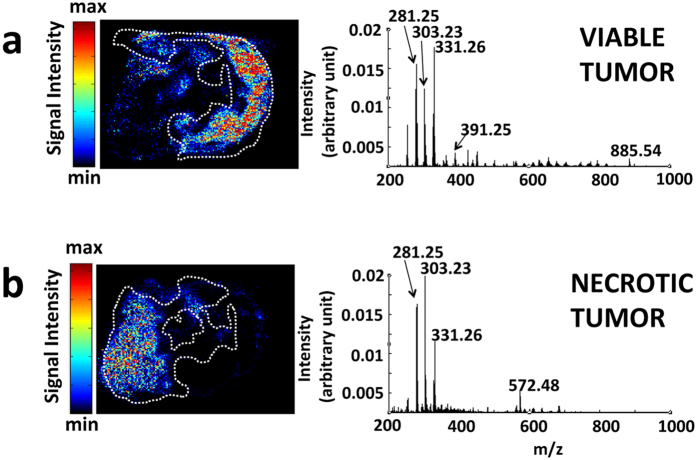
Spatially correlated regions from Non-Negative Matrix Factorization (NMF) analysis and their characteristic MS profile. The NMF analysis of the entire DESI-MS imaging dataset from which some ion images are given in Fig. 1 is presented alongside the characteristic spectra at each region. (**a**,**b**) The NMF analysis suggests two regions containing highly correlated MS spectra. The average spectra from each region produced through NMF analysis are also given. The borders marked with white dashed lines delineate areas of viable (**a**) and necrotic (**b**) cancer tissue from independent pathology. The correlation between NMF and pathology is reasonable but falls short of complete concordance.

DESI-MS images were recorded from 10 μm breast cancer tumor slices that were subsequently stained with Eosin and Haematoxylin (H&E) for pathology assessment. The slices contained both necrotic and viable cancer tissue, as revealed by H&E and Pan-cytokeratin (Pan-CK) staining. Pan-CK is a specific immunostain capable of identifying viable breast cancer cells with strong staining due to their epithelial origin (Fig. 1a). Based on the correlation between DESI-MS ion images and pathology, we identified the MS profile of the viable and the necrotic breast cancer (Fig. 1b,c). The DESI-MS profile of viable and necrotic cancer is quite different from the profile of healthy breast tissue shown in Fig. 1d. To corroborate the MS profiles of necrotic and viable cancer, we subjected the entire DESI-MS imaging dataset of the tumor slice to Non-negative Matrix Factorization (NMF) analysis to determine the areas of sample heterogeneity containing correlated mass spectral features. NMF does not use feedback from pathology to reveal sites of spectral heterogeneity, and is thought to remove bias introduced by pathology to “find” MS spectral features that may correlate with areas of anatomic or pathologic heterogeneity. Figure 2 shows the results of the NMF analysis, revealing two regions in the tumor that each contained distinct, highly correlated MS profiles, alongside the MS profiles that characterize them. Cross correlation with pathology results shown in Fig. 1 reveals that these regions are consistent with necrotic and viable cancer tissue, each possessing a unique MS profile as provided by the NMF analysis (Fig. 2). This cross correlation is required to give meaning to the segmentation provided by the NMF analysis. The MS profile of the necrotic and viable cancer tissue from NMF analysis is similar to the DESI-MS profiles of these two regions shown in Fig. 1b,c.

The DESI-MS ion images presented in Fig. 1a suggest a slight increase in the relative intensity of known breast cancer fatty acid (FA) biomarker ions of *m/z* 281.25 [FA(18:1)-H]^−^ (oleic acid) and 303.23 [FA(20:4)-H]^−^ (arachidonic acid)^11^,^12^,^22^ in the necrotic area. Unlike these two biomarkers, the ion of *m/z* 331.26 [FA(22:4)-H]^−^ (adrenic acid)^11^,^12^,^22^, which is also known to be a prominent breast cancer marker, is slightly less abundant in the necrotic center. Most strikingly, the necrotic tissue could be unambiguously characterized by the presence of the ion of *m/z* 572.48, tentatively assigned as [Cer(d34:1) + Cl]^−^ which is not abundant in the viable cancer region (Fig. 1a). Supplementary Figure S2 shows an enlarged view of the spectrum around 572.48 indicating its isotopic pattern along with limited MS/MS analysis performed to assign its identity. In addition, the ion of *m/z* 391.25 that commonly exhibits small abundance in viable cancer tissue is not present in the necrotic region at all (Fig. 1a). This ion is not currently assigned, and has also been observed in the MS analysis of non-necrotic human breast cancer biopsies by Agar and coworkers^12^. The observed *m/z* value, the theoretical mass, the error and the tentative assignment for each of the biomarker ion discussed above are listed in Supplementary Table S1. Figure 1a shows the DESI-MS images of the biomarker ions included in this table. As illustrated in Fig. 1a, the DESI-MS image of the ion of *m/z* 572.48 [Cer(d34:1) + Cl]^−^ clearly indicates the necrotic region identified by pathology. A combination of *m/z* 572.48 (for necrotic) and *m/z* 391.25 (for viable) MS ion images delineates the entire outline of the tumor slice (Fig. 1a).

Consistent with our results above that correlate the spatial distribution of [Cer(d34:1) + Cl]^−^ with necrotic areas, ceramides have been shown to accumulate during cell death, serving as a coordinator of stress response pathways^23^. More specifically, an accumulation of a variety of ceramides including [Cer(d18:1)(16:0) + Cl]^−^, which is a member of [Cer(d34:1) + Cl]^−^ family, has been reported in Jurkat apoptotic leukemia cell extracts using Electrospray Ionization Mass Spectrometry (ESI-MS)^24^. In a similar vein, an ion of *m/z* 572.7 has been noted in the MS spectra of necrotic Glioblastoma tumors resected from patients^25^. This lends further credence to potential clinical relevance of our findings in breast tissue that are currently based on xenograft models, also suggesting this ion may be a general marker for necrosis in tissues other than the breast. Consistent with our findings that phospholipid biomarker ions of the breast cancer exhibit changes in abundance during necrosis, a study of five different necrotic cancers (adenocarcinomic alveolar basal epithelial cells, colon carcinoma, pancreatic and lung cancers as well as glioblastoma-astrocytoma) with Matrix Assisted Laser Desorption Ionization Mass Spectrometry (MALDI-MS) reports alterations in the relative abundance of sodium adducts of a number of phospholipids during necrosis^26^. Similar alterations in phospholipid abundances have also been reported with MALDI-MS in hypoxic breast cancer tumors presumably due to altered lipid metabolism cascades^27^.

**Figure 3:**
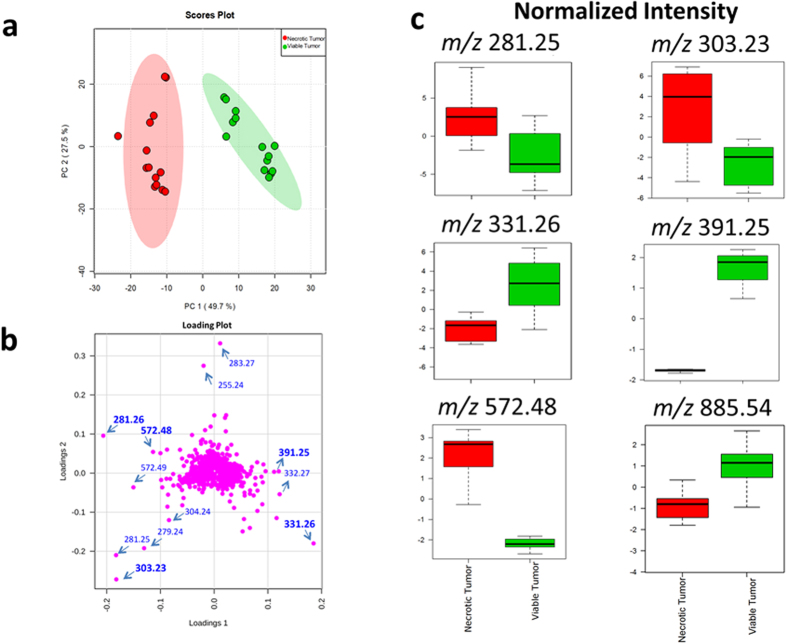
Principal Component Analysis (PCA) of the necrotic breast cancer. (**a**) PCA scores plot shows the statistical discrimination between MS profile of the necrotic and viable cancer tissue within the same tumor. The PCA scores for components 1,2 are determined from the entire spectra (i.e. all *m/z* values). The highlighted area for each component corresponds to a confidence interval of 95%. (**b**) PCA loading plot illustrating the ions that contribute strongly to statistical separation between the viable and necrotic cancer profiles. (**c**) The box plots indicating changes in ion intensity (normalized to TIC) of biomarker ions predicted by NMF analysis to contribute most significantly to the statistical discrimination between necrotic and viable tissue (Supplementary Table S1). These plots are available for the variables subjected to PCA where the bottom and top of each box represent the 25th and 75th percentile (the lower and upper quartiles, or Q1 and Q3), and the band in the middle shows the 50th percentile (the median or Q2). The upper whisker represents the smaller of the maximum variable value and Q3 + 1.5 IQR (Interquantile Range), and the lower whisker is located at the larger of the smallest variable value and Q1–1.5 IQR.

To examine the reproducibility of our findings above, we subjected the average spectra of necrotic and viable cancer tissue from 7 DESI-MS images of serial sections from the same tumor analyzed in Fig. 1 to Principal Component Analysis (PCA). We selected two Regions of Interest (ROI) from each necrotic and viable subregion and included 14 data points in the evaluation with PCA. Supplementary Figure S3 shows the DESI-MS images of all tissue sections used in the PCA, along with their respective H&E images used for pathology assessment. The spatial distribution of the ion of *m/z* 572.48 in all sections matched the pathology, indicating necrotic areas (Supplementary Figure S3). The spatial distribution maps for other ions shown in this figure also matched the single slice representative results shown in Fig. 1. The DESI-MS data were thus consistent within sections prepared from the same tumor specimen. Figure 3 shows the PCA scores plot and box plots that illustrate changes during necrosis in the abundance of biomarker ions listed in Supplementary Table S1. Changes in median ion abundance values between necrotic and viable regions are consistent with what is reported in Fig. 1. However, the large variations seen in ion abundances from the necrotic center, shown in box plots, must be taken into consideration to evaluate the statistical significance. The PCA scores plot (Fig. 3a) suggests a clear statistical discrimination of necrotic from viable breast cancer based on the MS profile of their lipid contents with ion abundance changes between viable and necrotic cancer tissue as illustrated in Fig. 1 through pathology guided assessment of ion images. This pattern additionally validates the MS profiles suggested by unsupervised NMF analysis in Fig. 2. A closer inspection of the concordance between NMF results and pathology reveals that although the NMF analysis predicted the necrotic region with reasonable accuracy, the areas depicted by this method to be necrotic do not account for the entire area of necrosis revealed by pathology. Here, DESI-MS imaging of the ceramide ion of *m/z* 572.48 provides the closest match to histology for the determination of the necrotic areas. Likewise, NMF fell short of mapping the viable cancer tissue in its entirety. DESI-MS imaging of the ion of *m/z* 391.25 provided the most robust means of mapping the viable cancer region with the closest match to pathology (Fig. 1). Currently, pathology feedback is required to interpret DESI-MS images. Further work is required to increase the robustness of unsupervised methods towards a closer match with pathology results. Nevertheless, MS image analysis methods such as NMF that do not use feedback from pathology will prove useful in stand-alone MS-based molecular pathology platforms offering accelerated diagnoses. NMF was able to successfully predict biomarker ions of *m/z* 391.25 and *m/z* 572.48 for viable and necrotic cancer tissue respectively. Many investigators use PCA to tease out *m/z* values that contribute to statistical separation between samples. This is achieved through using the so-called loading plots that indicate the relative rank of each variable *m/z* in how strongly they contribute to discrimination between samples. In this study, we used PCA as a secondary verification tool to assess the statistical significance of the *m/z* values reported by the combination of NMF and pathology to populate the average DESI-MS profile of necrotic and viable cancer tissue (Fig. 2). The PCA loading plot (Fig. 3b) indicates that the same ions suggested by NMF to populate the average DESI-MS profiles of necrotic and viable cancer regions are indeed the ones that strongly contribute to the statistical separation seen.

**Figure 4:**
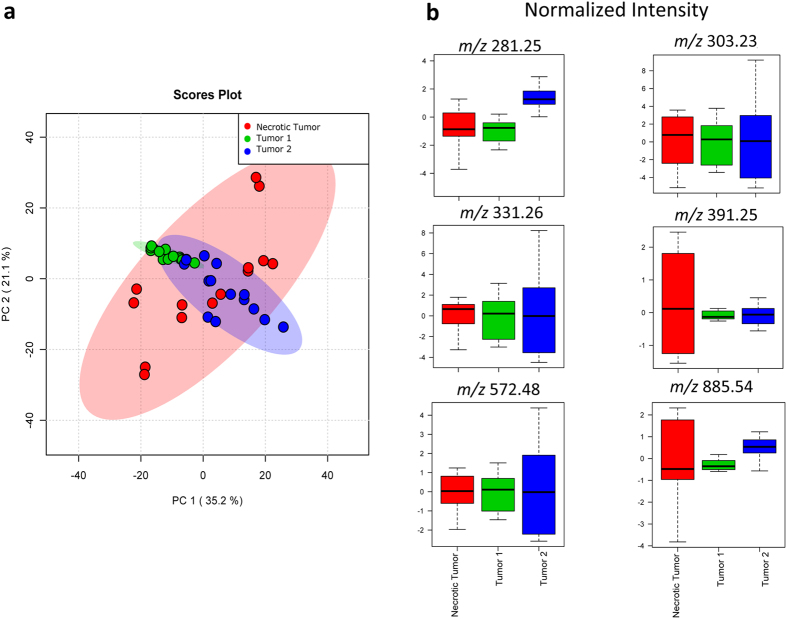
Principal Component Analysis (PCA) of tumors 1,2 and the necrotic tumor. (**a**) PCA scores plot does not suggest statistical separation between MS profiles. The highlighted area for each component corresponds to a confidence interval of 95%. (**b**) The box plots of the biomarker ions characterizing cancer show within the error similar values of normalized ion intensity for all markers, strongly suggesting that tumors 1,2 are necrotic.

A potential caveat is that the profile described above is based on teachings from 7 tissue slices from a single tumor specimen (referred to as the “training” tumor). To address this, we examined whether the necrosis lipid MS profile above was broadly representative and could be utilized to identify independent necrotic tumors in a blind test. We produced subcutaneous xenograft tumors of breast cancer from LM2-4 cell line in 3 additional, independent SCID mice and used a total sample size of 30 tissue slices in this study. In combination with two randomly chosen regions of interest from each tissue sample, the findings reported in this manuscript are consistent across 60 data points. We harvested the tumors after 2–3 weeks to increase the chance of widespread necrosis, and subjected tissue slices to DESI-MS imaging and post-DESI-MS staining with H&E for pathology assessment. Figure 4a shows the PCA scores plot of the average MS spectra from tumors 1 and 2 (blind test) along with those of the “training” necrosis dataset described in Fig. 3. PCA showed no statistical separation between DESI-MS profiles of the tumors 1,2 and the necrotic tumor of the “training” dataset, strongly implicating that tumors 1,2 were highly necrotic. Further supporting this, the box plots of the ion abundances show identical normalized intensities for all breast cancer biomarker ions between tumors 1,2 and the “training” dataset (Fig. 4b). Supplementary Figures S4,5 show the DESI-MS images of tumor 1,2 slices used in the PCA assessment, along with the H&E images completed after the PCA blind test. As seen here, H&E staining and feedback from the pathologist involved in the blind study confirmed that tumors 1,2 were largely necrotic. Tumor 1 is entirely necrotic as no viable cancer biomarker marker ion of m/z 391.25 was detected (Supplementary Figure S4). Tumor 2 contained small areas of viable cancer on the periphery of the largely necrotic core (Supplementary Figure S5). Consistent with this, the DESI-MS images of these tumors confirm the presence of the ion of *m/z* 572.48, further supporting widespread necrosis (Supplementary Figures S4,5).

**Figure 5:**
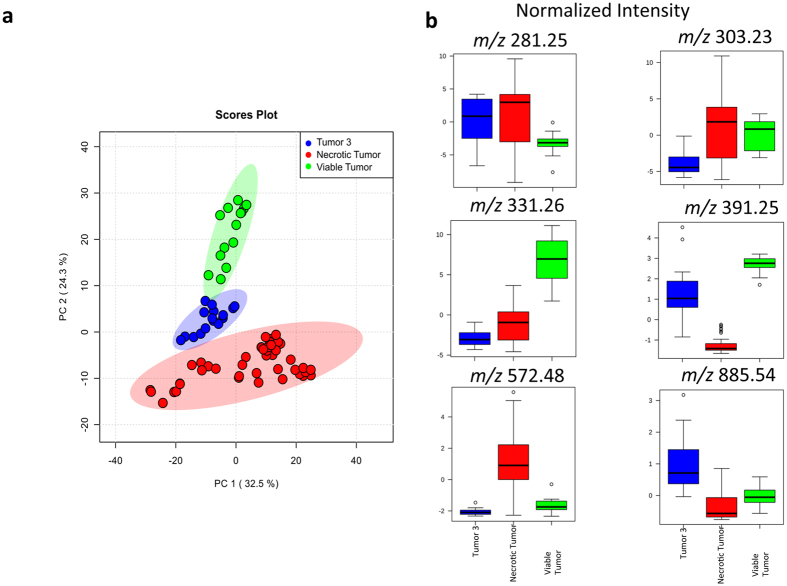
Principal Component Analysis (PCA) of tumor 3 and the necrotic and viable tumor. (**a**) PCA scores plot suggests that the MS profile of the tumor 3 possesses features that are intermediary between necrotic and viable cancer regions from training tumor and tumors 1,2. The highlighted area for each component corresponds to a confidence interval of 95%. (**b**) Consistent with the results of the scores plot, the box plots of the biomarker ions characterizing cancer show intermediate normalized ion intensity values compared to necrotic and viable cancer tissue. The pathology assessment (Supplementary Figure S7) confirms that tumor 3 was in the process of becoming fully necrotic.

Figure 5 shows the PCA of the third tumor subjected to the blind assessment (See Supplementary Figure S6 for DESI-MS and H&E images of the tumor 3 slices). Here we show a 3-component PCA that used all viable and necrotic imaging datasets from independent tumors for comparison with tumor 3 data. This figure illustrates the statistical separation between necrotic and viable cancer data points from all tumors examined in this study, further suggests that the representative results shown in Fig. 1 are consistent across the independent samples examined. The latent features in tumor 3 exhibited statistical distributions that were intermediate to viable and necrotic cancer tissue using data from all independent tumors examined in this study (Fig. 5a). Consistent with this, the box plots indicated biomarker ion abundances that were intermediate between viable and necrotic tissue (Fig. 5b). A closer examination of the pathology for this tumor indicated that its cancer cells were in the process of full necrotic transformation. The membrane morphology of the cancer cells in tumor 3 had necrotic characters (Supplementary Figure S7) yet a small amount of the viable cancer biomarker ion of *m/z* 391.25 was detected in this tissue (Supplementary Figure S6). The Supplementary Figure S7 shows an enlarged view of the H&E image to better illustrate this point. Consistent with this as shown in Supplementary Figure S6, the necrosis biomarker ion of *m/z* 572.48, also present in this tissue, had a uniform distribution over the surface of the tumor 3 sample, albeit at a lower general abundance compared to the necrotic tumors 1,2 which were imaged under identical DESI-MS conditions.

**Figure 6:**
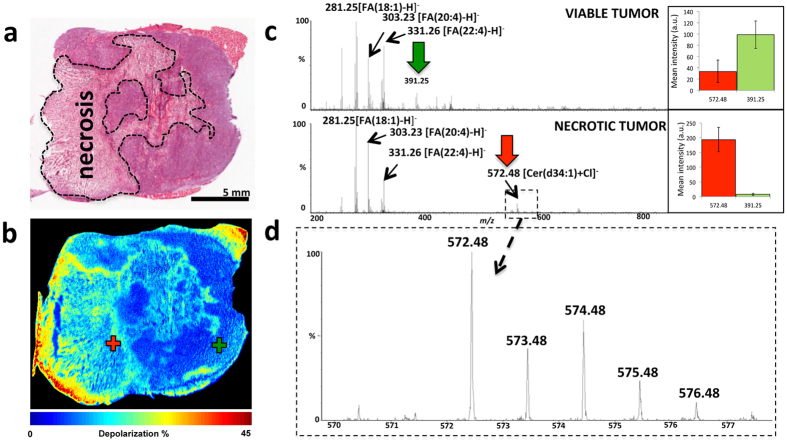
Wide-field tissue polarimetry allows targeted acquisition of DESI-MS profiles from necrotic cancer regions. (**a**) The H&E image of a 10 μm tissue slice. (**b**) The polarimetric image of the tissue slice shown in (**a**). Increased tissue depolarization reveals necrotic areas. (**c**) Single scan MS profiles from two spots within viable and necrotic centers (marked with the ‘+’ symbol on the polarimetric image). These spectra were collected within 1 s of acquisition and contain the characteristic MS profiles of the necrotic and the viable cancer regions. The abundance of the viable/necrotic cancer biomarker ions using 150 data points (75 viable, 75 necrotic), each averaged over 4 pixels, is shown. The bar graphs display mean ion abundance or intensity in arbitrary units (y-axis) ±1 SD (standard deviation) of the distribution. For necrotic site, the abundances of *m/z* 572.48 and *m/z* 391.25 were 190 ± 40 and 7 ± 3, respectively. For the viable site, the abundances of *m/z* 572.48 and *m/z* 391.25 were 35 ± 20 and 100 ± 25, respectively. (d) Zoomed image of *m/z* 572.48 highlighting the isotopic pattern of this ion.

The DESI-MS images shown in Fig. 1 took an average of ~5 hours to collect at 100 μm spatial resolution. This analysis time is very long compared to intraoperative histology, which could be completed in 10–20 min. We used guidance from wide-field tissue polarimetry to target the DESI-MS profiling of necrotic regions allowing analysis in minutes, faster than currently possible with histology methods^15^. Figure 6a shows the resultant polarimetric image of a 10 μm tumor slice revealing areas of polarimetric heterogeneity that correlate well with necrotic regions from pathology (Fig. 6b). Necrotic transformation in the tissue was accompanied by reduced depolarization detectable with Mueller matrix tissue polarimetry^28^. Targeting the DESI spray tip using a polarimetry image to select areas marked with crosses in Fig. 6a allowed acquisition of the MS spectrum of the necrotic breast cancer within a single MS scan in ~1 s. This spectrum contained all key biomarker ions that describe necrotic breast cancer (Supplementary Table S1). This combination leads to an overall polarimetry/MS analysis time of slightly less than 2 minutes to detect necrosis in a breast cancer slice. For rapid point profiling using tissue slices, polarimetry can improve the overall efficiency by 2 orders of magnitude (specifics dependant on the size of the imaged area^15^). To further validate the reproducibility of polarimetric guidance we selected 150 points over the necrotic and viable regions (n = 75 each) and quantified the abundance of the necrotic and viable cancer biomarker ions in these areas. The bar graphs shown in this figure suggest that the tissue areas assigned by polarimetry to be necrotic indeed possessed a statistically higher abundance of the necrotic cancer biomarker ion of *m/z* 572.48. Likewise, the viable cancer centers contained an abundance of the viable cancer biomarker ion of *m/z* 391.25.

To further lower the analysis time, we coupled DESI-MS analysis using single MS scans with the most rapid means of sample preparation, which uses tissue smears prepared in less than 1 min^9^,^29^,^30^. The preparation of a smear may take less than 30 seconds. Supplementary Figure S8 shows a representative single scan MS profile (~1 s acquisition) acquired from a random position on the surface of a necrotic breast cancer tissue smear prepared on porous polytetrafluoroethylene (PTFE) surface, along with enlarged views of DESI-MS spectra around *m/z* 572.48 from 10 randomly selected positions. We were able to detect necrosis marker *m/z* 572.48 in all of these spectra. A recent study from our group has investigated the further utility of smear samples for MS profiling, including the limit of detection and reproducibility^31^.

In this study we report the lipid DESI-MS profile of necrotic breast cancer with potential applications in identifying necrotic tumors solely on the basis of their lipid MS profile. To allow faster than currently possible detection of necrosis with conclusive MS fingerprinting, wide-field polarimetry can guide DESI-MS imaging to sites of polarimetric heterogeneity. This workflow allows rapid necrotic cancer identification via targeted DESI-MS profiling as shown in Fig. 6. Guided DESI-MS imaging and boundary assessment for necrotic centers is also possible using polarimetry images. Furthermore, the lipid MS profile could be used to rapidly determine necrosis using tumor smears. The concordance of section/smear profiles observed herein and by other investigators^9^,^29^,^30^ is likely to create a paradigm shift in utilizing tissue smears for rapid intraoperative cancer diagnosis with DESI-MS profiling. Our preliminary results presented in this manuscript are thus promising for future development of a clinical assay for rapid identification of necrosis to be validated using a larger number of independent patient samples, to account for intrinsic biological variations between human samples.

## Additional Information

**How to cite this article**: Tata, A. *et al*. Rapid Detection of Necrosis in Breast Cancer with Desorption ElectroSpray Ionization Mass Spectrometry. *Sci. Rep.* **6**, 35374; doi: 10.1038/srep35374 (2016).

## Supplementary Material

Supplementary Information
