# Supplementary material for: Rapid Detection of Necrosis in Breast Cancer with Desorption Electrospray Ionization Mass Spectrometry
Source: Sci Rep. 2016 Oct 13;6:35374. doi: 10.1038/srep35374 (PMC5062153; doi:10.1038/srep35374)
Supplement: Supplementary Information [file srep35374-s1.pdf]

Supplementary Information

Rapid Detection of Necrosis in Breast Cancer with Desorption Electrospray Ionization Mass Spectrometry

Alessandra Tata[a,*], Michael Woolman[a,*], Manuela Ventura[a], Nicholas Bernards[a], Milan Ganguly[b], Adam Gribble[c], Bindesh Shrestha[h], Emma Bluemke[a], Howard J. Ginsberg[a,d,e], Alex Vitkin[c,f,g], Jinzi Zheng[a, i], and Arash Zarrine-Afsar[a,c,d,e,**]

[a]Techna Institute for the Advancement of Technology for Health, University Health Network, Toronto, ON, M5G-1P5, Canada
[b]STTARR Innovation Center, Princess Margaret Cancer Center, 101 College Street, Toronto, ON M5G 1L7
[c]Department of Medical Biophysics, University of Toronto,101 College Street Suite 15-701, Toronto, ON, M5G 1L7, Canada
[d]Department of Surgery, University of Toronto, 149 College Street, Toronto, ON, M5T-1P5, Canada
[e]Keenan Research Center for Biomedical Science, Li Ka Shing Knowledge Institute, St. Michael's Hospital, 30 Bond Street, Toronto, ON, M5B-1W8, Canada
[f]Department of Radiation Oncology, University of Toronto, 610 University Avenue, Toronto, Ontario M5G 2M9, Canada
[g]Division of Biophysics and Bioimaging, Ontario Cancer Institute, University Health Network, 610 University Ave, Toronto, ON M5G 2M9
[h] Waters Corporation, 34 Maple Street, Milford, MA 01757, USA
[i]Institute of Biomaterials and Biomedical Engineering, University of Toronto, 164 College Street, Toronto, ON M5S 3G9
[*]These authors contributed equally to this work
[**]Correspondence to arash.zarrine.afsar@utoronto.ca

Table of content

Figure S1……………………………Page 2
Figure S2……………………………Page 3
Figure S3……………………………Page 5
Figure S4……………………………Page 5
Figure S5……………………………Page 6
Figure S6……………………………Page 7
Figure S7……………………………Page 8
Figure S8……………………………Page 9
Figure S9……………………………Page 10
Table S1………………………………Page 11
References…………………………..Page 12

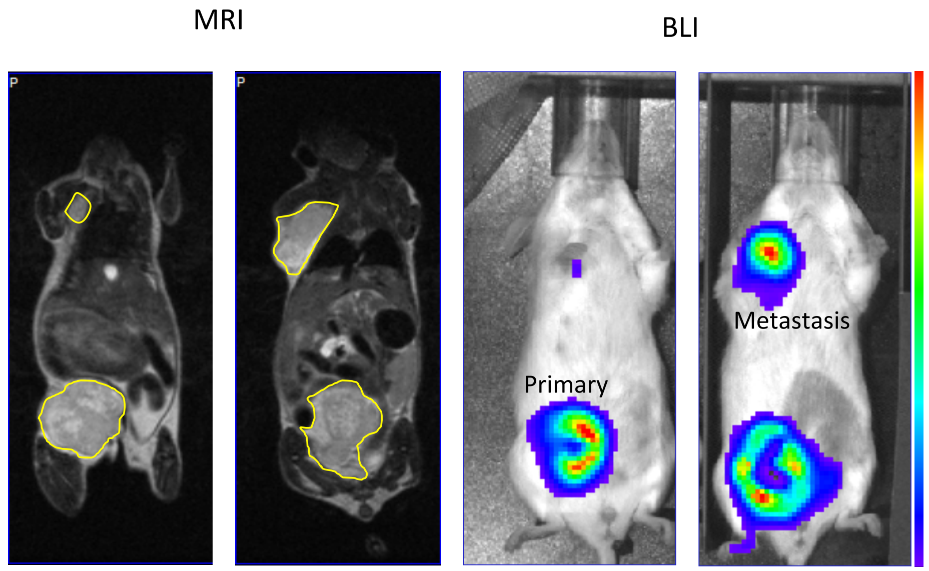

Figure S1. ***In vivo*** **imaging of Tumors.** The MRI (Left) and the BLI (Right) images of two representative mice containing metastatic cancer in the lymph nodes along side primary tumors regrown at the inoculation site.

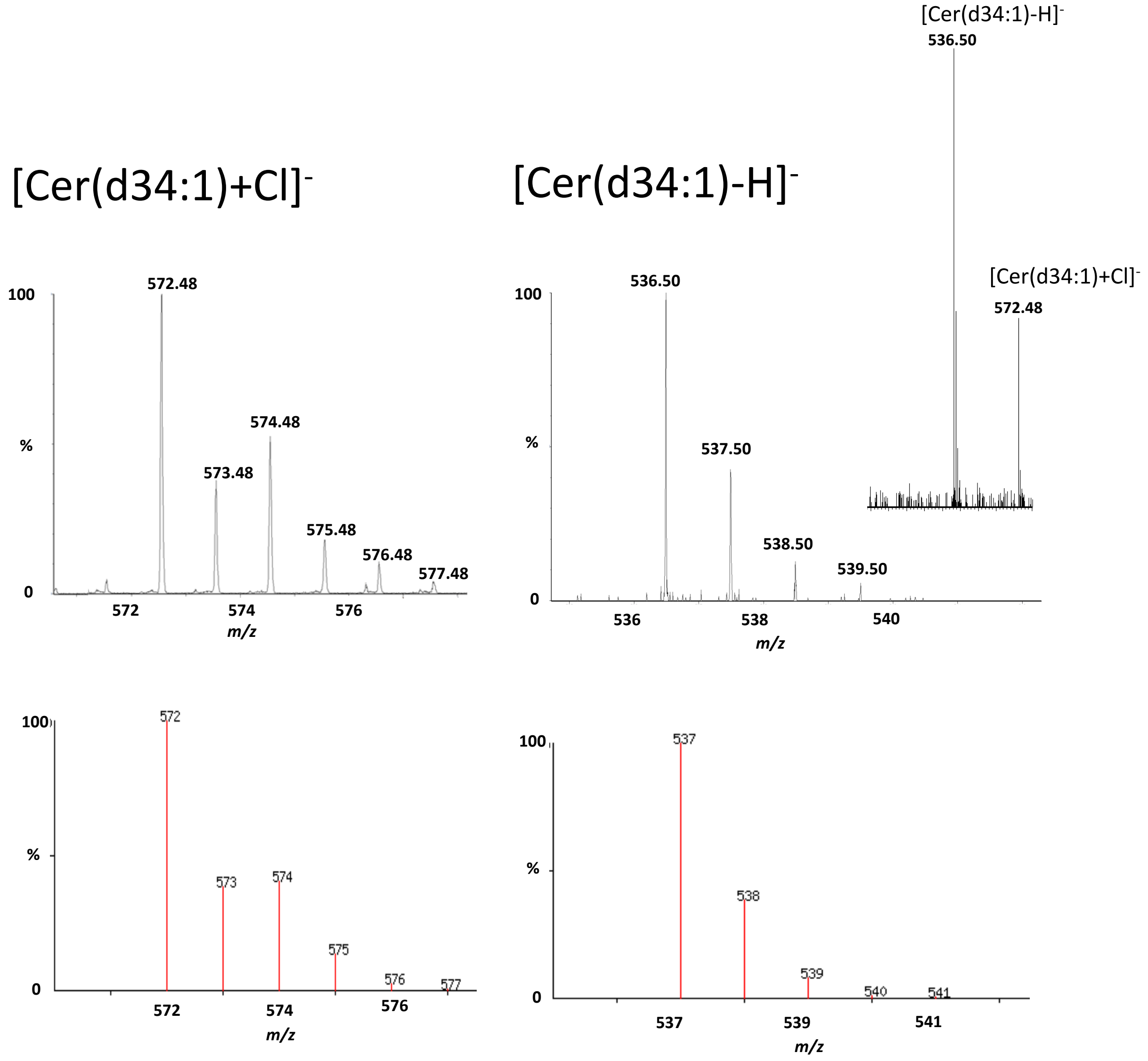

Figure S2. The assignment of *m/z* 572.48 as [Cer(d34:1)+Cl]$^{-}$ based on isotopic pattern distribution. The acquired spectrum (zoomed view) in the vicinity of *m/z* 572.48 (top) is shown alongside the predicted isotopic pattern of this ion. The MS/MS fragmentation produced loss of chlorine and a daughter ion with *m/z* of 536.50 whose isotopic distribution pattern matched that of [Cer(d34:1)-H]$^{-}$. We were unable to further fragment [Cer(d34:1)-H]$^{-}$ using untargeted LC-MS/MS method. As illustrated in the Supplementary Table S1, through a database search using Lipidmaps isobaric species of Cer(d18:1)/(16:0), Cer(d16:1)/(18:0), Cer(d14:1)/(20:0) are consistent with this assignment, out of which in the literature chlorinated adducts of C16 ceramides such as [Cer(d18:1)(16:0)+Cl]$^{-}$ have been shown to accumulate in dying cells [1,2]. Such adducts are stable products, and have also been seen in MS analysis of standard compound ceramides[3].

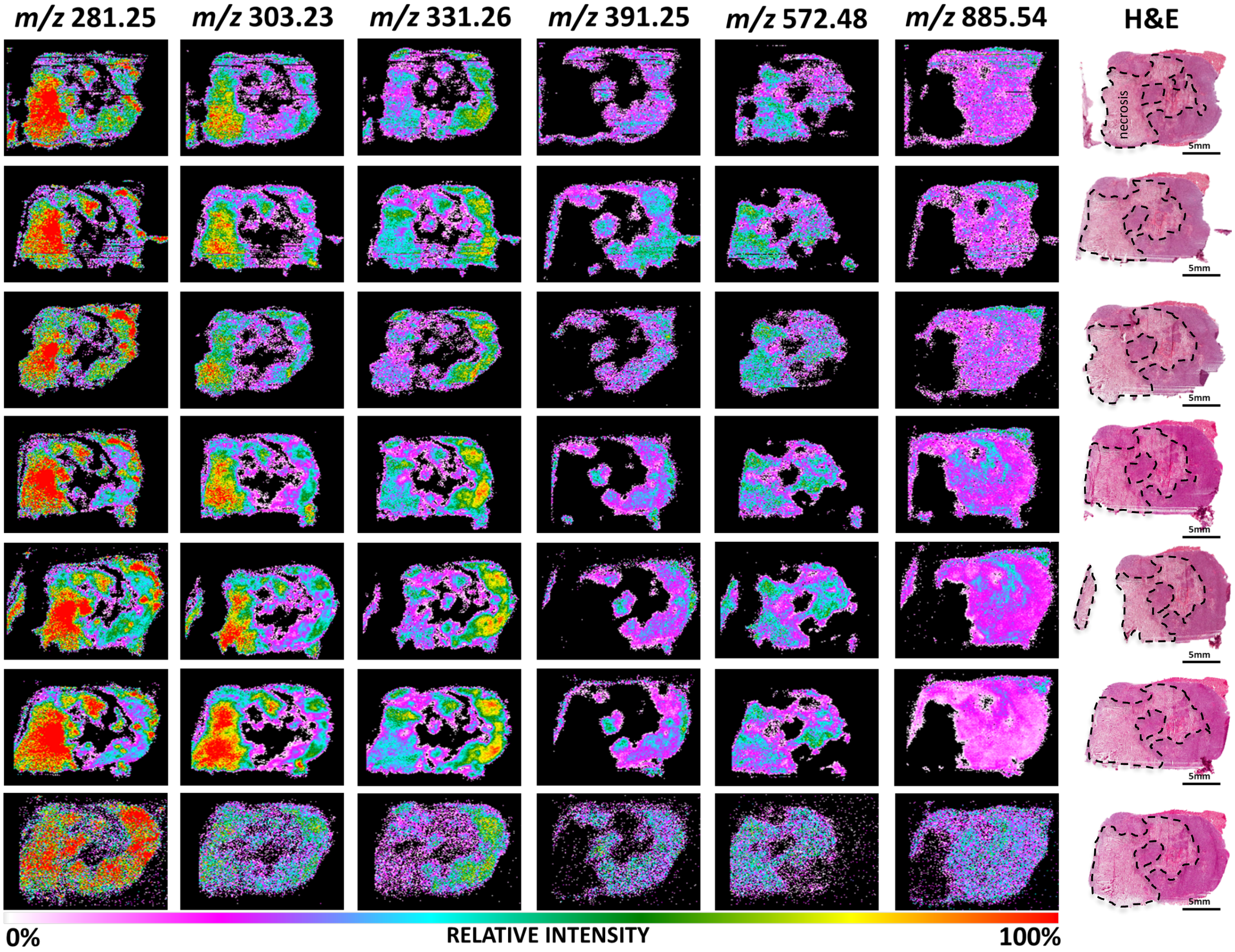

Figure S3. The DESI-MS ion images of the consecutive slices of the "training" tumor dataset containing necrotic and viable cancer sites. The H&E image of the same slice is also given. The ion images for *m/z* values contributing to the statistical separation between necrosis and viable cancer sites are presented. Consistent with the results shown in Fig. 1 the image of the ion of *m/z* 572.48 matches the necrotic area (from H&E).

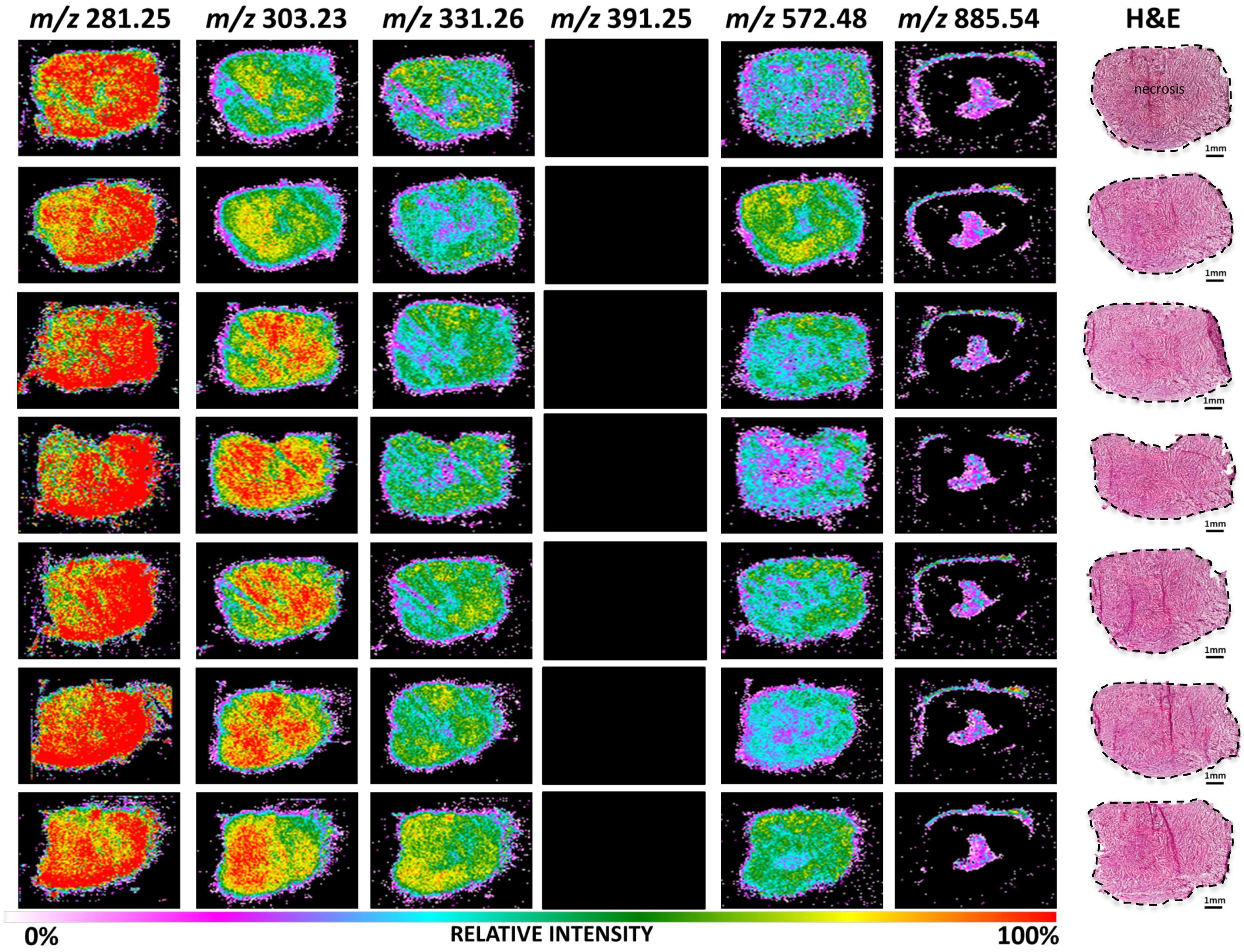

Figure S4. The DESI-MS ion images of the consecutive slices of Tumor 1. The H&E image of the same slice is also given. The ion of *m/z* 391.25 prevalent in the viable cancer site was not detected in this sample. The necrosis marker ion of *m/z* 572.48 is seen to be present uniformly across the entire surface of the section, indicating widespread necrosis.

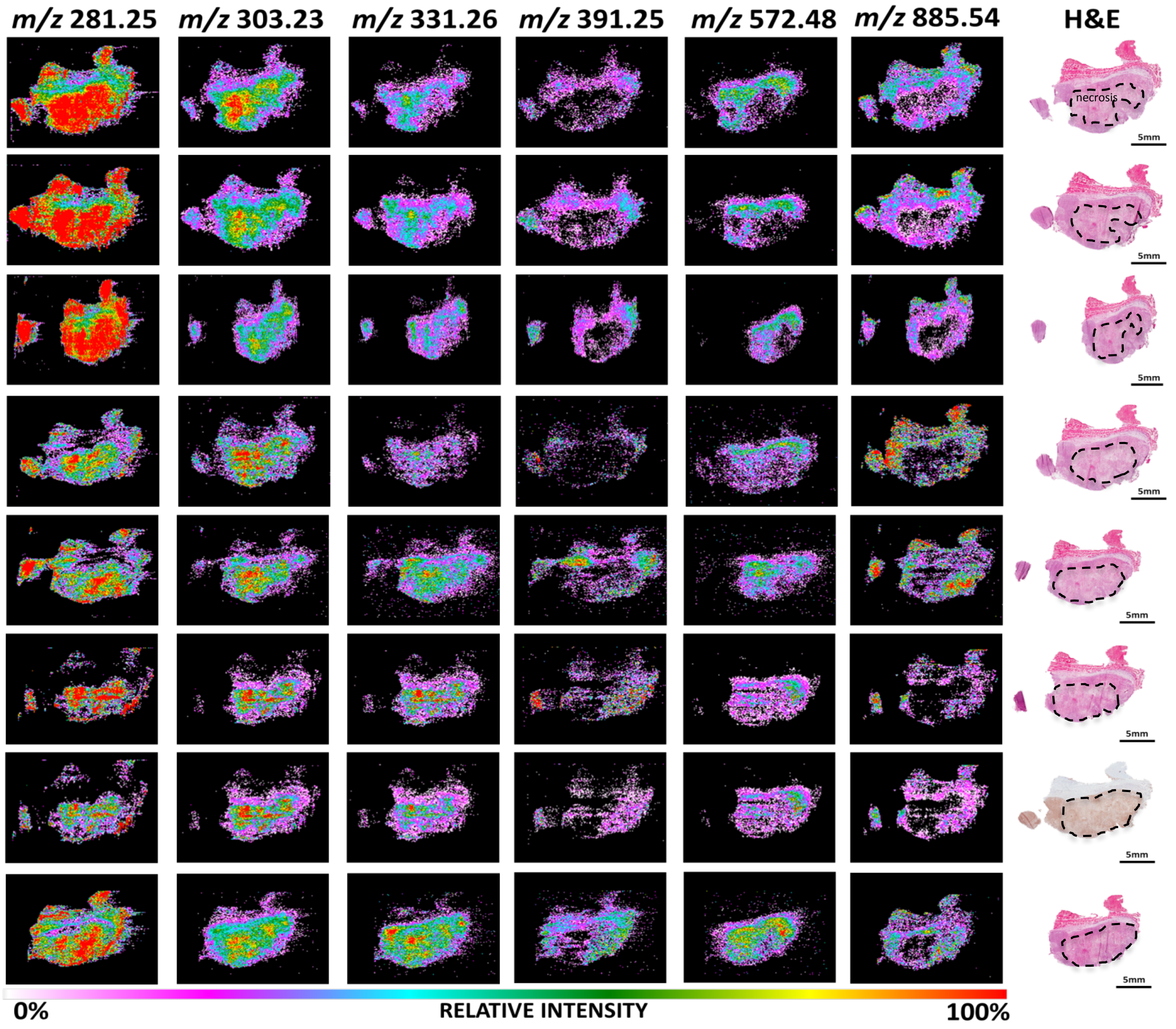

Figure S5. The DESI-MS ion images of the consecutive slices of Tumor 2. The H&E image of the same slice is also given. The H&E image is replaced with the Pan-CK stained image for panel 7. As with the “training” tumor dataset (Fig. 1, Supplementary Figure S3), the necrosis marker ion of *m/z* 572.48 is present in the areas that from pathology (H&E) are known to be necrotic.

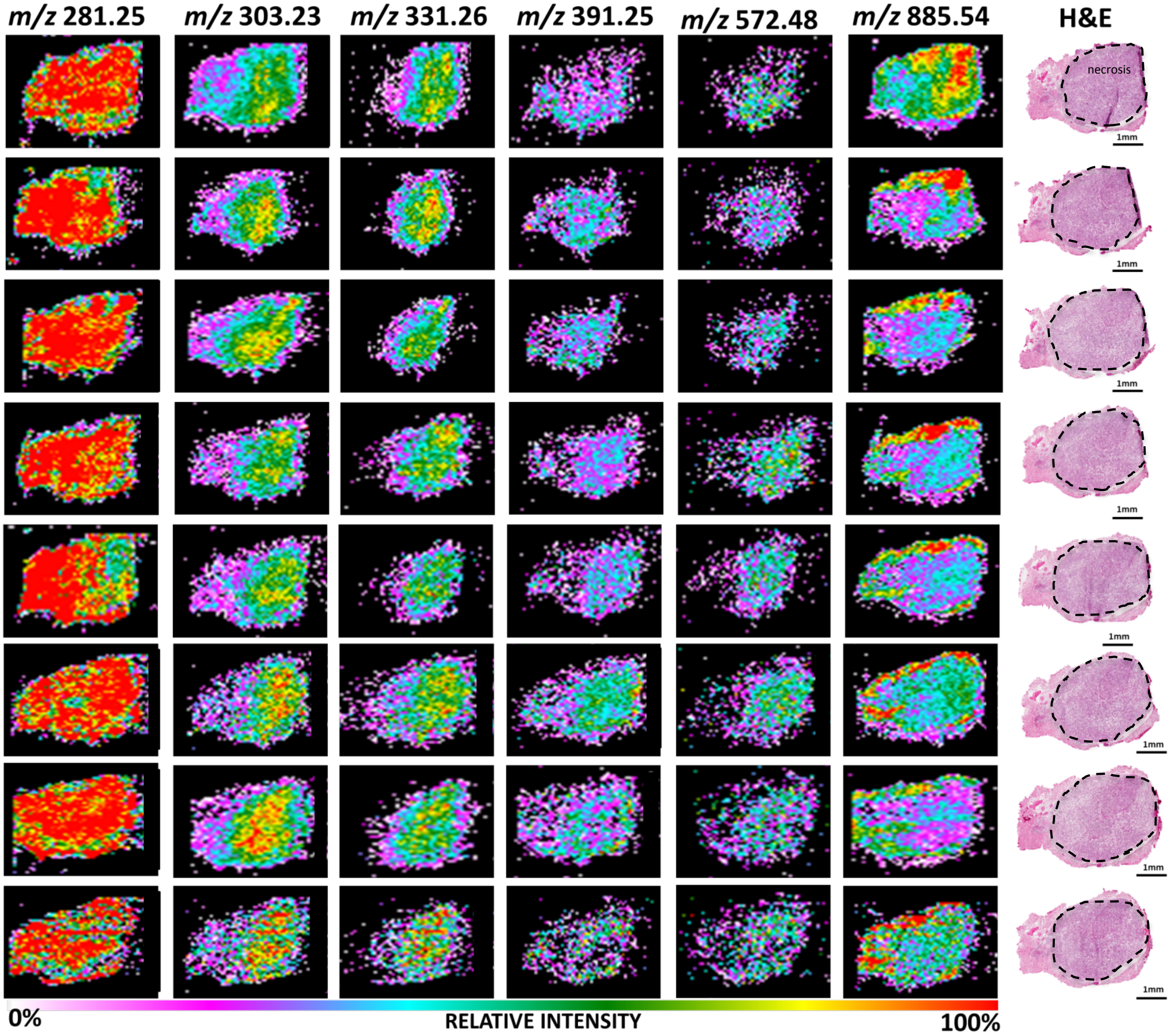

Figure S6. The DESI-MS ion images of the consecutive slices of Tumor 3. The H&E image of the same slice is also given. The marker ion of viable cancer (*m/z* 391.25) and the marker ion of necrotic breast cancer (*m/z* 572.48) are both present across the entire surface of the section.

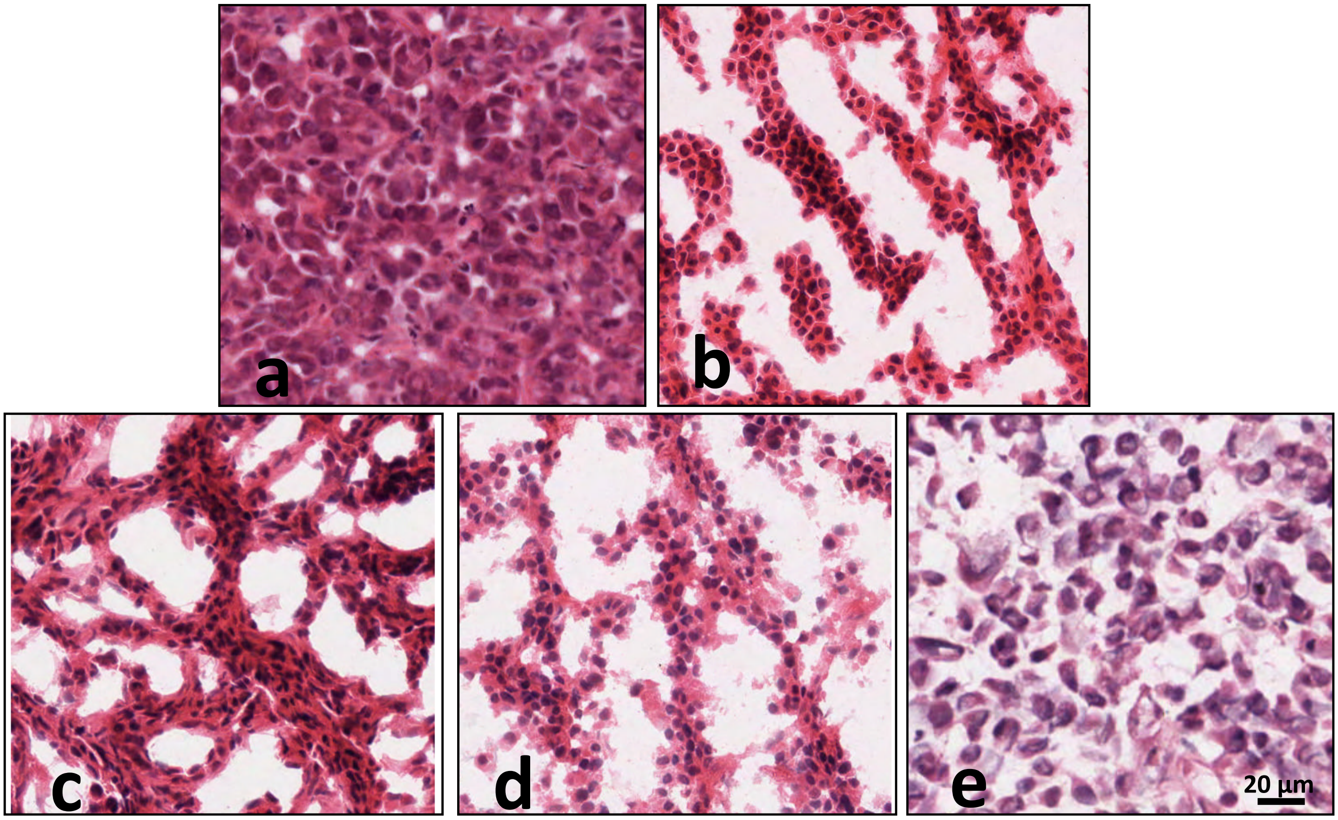

Figure S7. Pathology assessment of all tumors used in this study using H&E staining and microscopy. (a) The viable region of the “training” tumor. (b) The necrotic region of the “training” tumor. (c) Tumor 1. (d) Tumor 2. (e) Tumor 3.

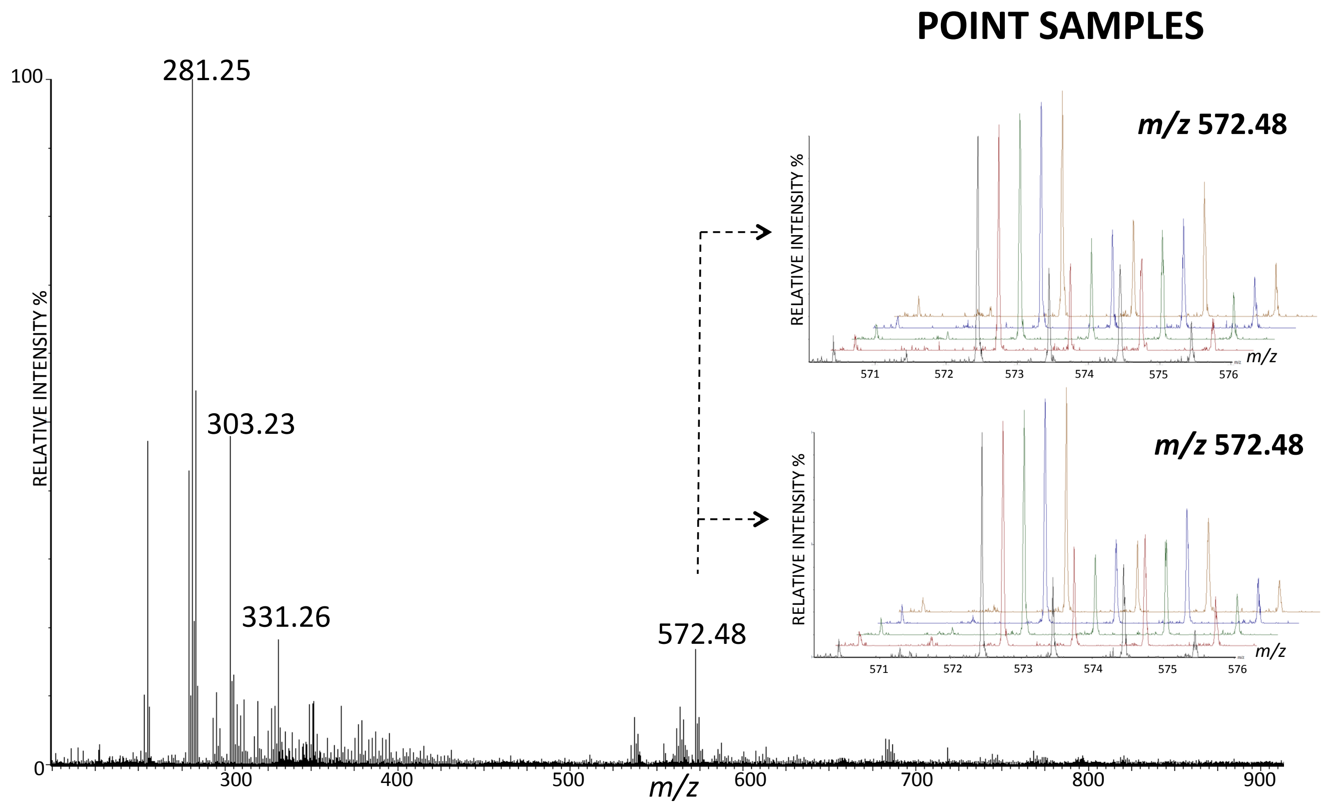

Figure S8. Single MS scans of the necrotic tumor smear on porous PTFE. A 1 mm thick porous PTFE foil (Berghof), cut to the same dimension of a commercial glass slide to fit in the bed of the DESI-MS moving stage, using a second slide to flatten and spread the tissue, as performed within the workflow for rapid intraoperative pathology.The necrosis biomarker ion of *m/z* 572.48 (highlighted region) is detected in all random positions on the surface of the smear examined. Here we show enlarged views of 10 single scan MS profiles (~1 s acquisition) acquired from random positions across the smear on porous PTFE. In all of these spectra we were able to detect necrosis marker *m/z* 572.48. The smear preparation took 30 s. Therefore, in combination with the marker we have discovered, necrotic breast cancer can be identified from tissue smears in less than 1 min of overall preparation and analysis time.

m/z observed by MS	Theoretical mass	Error (Δ ppm)	Assignment	Type of ion
281.2467	281.2486	-6.75	Oleic acid, FA (18:1)	$[M-H]^-$
303.2299	303.2330	-10.22	Arachidonic acid, FA (20:4)	$[M-H]^-$
331.2596	331.2643	-14.18	Adrenic acid, FA(22:4)	$[M-H]^-$
391.2501	N/A	N/A	N/A	N/A
572.4786	572.4815	-5.06	**Cer(d34:1)**<br>Cer(d18:1)/(16:0)<br>Cer(d16:1)/(18:0)<br>Cer(d14:1)/(20:0)	$[M+Cl]^-$
885.5450	885.5499	-5.53	PI(18:0)(20:4)	$[M-H]^-$

Table S1. The assignment of *m/z* values and lipid identification. The assignments are provided by MS/MS analysis, isotopic pattern match, and accurate mass as described in the experimental section. Standard lipid nomenclature [4] is used, where the length of the aliphatic chain (in number of carbon atoms) : the number of double bonds are given for fatty acid (FA), phosphatidylinositol (PI) or ceramide (Cer). Known isobaric examples of $[Cer(d34:1)+Cl]^-$ from Lipidmaps database are given. In order to increase the confidence of biomarker identification, lipid extracts of xenograft tissue were analyzed using liquid chromatography (ACQUITY UPLC, Waters Corporation, Milford MA, USA) with mass spectrometry (SYNAPT G2Si, Waters Corporation, Milford MA, USA) using ion mobility-assisted data-independent analysis or $HDMS^E$ workflow. Briefly, lipid extracts were prepared by adding 150 µL of water, 190 µL of methanol and 370 µL of chloroform to ~ 100 mm^3 of a tissue. After 2 minutes of vortexing, the extract was centrifuged for 5 minutes at 13000 rpm. The apolar solution at the bottom layer was extracted into a tube and centrifuged again for 2 minutes at 13000 rpm to separate any remaining water. A dry nitrogen stream was then used to evaporate the solvent. The dried lipid extract was stored in sealed container at -20 °C until used. The lipid extract was re-suspended in 200 µL isopropanol/acetonitrile/water (2/1/1) solvent mixture for UPLC analysis using high mechanical stability column with a trifunctional C18 alkyl phase bonded at a ligand density promoting polar compound retention, as well as, compatible aqueous mobile-phase (ACQUITY UPLC HSS T3 column, 100Å, 1.8 µm, 2.1 mm X 100 mm). After chromatographic separation of 1 µL of extract, the sample was ionized by electrospray and analyzed by a mass spectrometer. The lipid ions were fragmented in the transfer T-Wave cell in $HDMS^E$ mode, which acquires mass spectra in both low and high collision energies. Precursor and product ions are formed during alternating low energy and high collision energy acquisition, respectively.

References

1 Kanto, T., Kalinski, P., Hunter, O. C., Lotze, M. T. & Amoscato, A. A. Ceramide mediates tumor-induced dendritic cell apoptosis. *Journal of immunology* **167**, 3773-3784 (2001).

2 Thomas, R. L., Jr., Matsko, C. M., Lotze, M. T. & Amoscato, A. A. Mass spectrometric identification of increased C16 ceramide levels during apoptosis. *The Journal of biological chemistry* **274**, 30580-30588 (1999).

3 Hsu, F. F. & Turk, J. Characterization of ceramides by low energy collisional-activated dissociation tandem mass spectrometry with negative-ion electrospray ionization. *J Am Soc Mass Spectrom* **13**, 558-570, doi:10.1016/S1044-0305(02)00358-6 (2002).

4 Fahy, E. *et al.* Update of the LIPID MAPS comprehensive classification system for lipids. *J Lipid Res* **50 Suppl**, S9-14, doi:10.1194/jlr.R800095-JLR200 (2009).
